# Online Physical Exercise and Group Sessions to Increase and Maintain Physical Activity in Individuals with Type 2 Diabetes: A Single-Arm Feasibility Study

**DOI:** 10.3390/ijerph20042893

**Published:** 2023-02-07

**Authors:** Sofie Rath Mortensen, Mathilde Espe Pedersen, Søren T. Skou, Mathias Ried-Larsen

**Affiliations:** 1 The Research Unit for Exercise Epidemiology, Centre of Research in Childhood Health, Department of Sports Science and Clinical Biomechanics, University of Southern Denmark, 5230 Odense M, Denmark; 2 The Research Unit PROgrez, Department of Physiotherapy and Occupational Therapy, Naestved-Slagelse-Ringsted Hospitals, 4200 Slagelse, Denmark; 3 Centre for Physical Activity Research, Rigshospitalet, 2100 København Ø, Denmark; 4 Research Unit for Musculoskeletal Function and Physiotherapy, Department of Sports Science and Clinical Biomechanics, University of Southern Denmark, 5230 Odense M, Denmark

**Keywords:** Type 2 diabetes, physical activity, online physical exercise, accelerometer, feasibility studies, eHealth, wearables

## Abstract

Current physical activity interventions for individuals with Type 2 diabetes do not accommodate the needs of the individual in terms of content, time, and location. The aim of this study was to evaluate the feasibility and acceptability of an 8-week high intensity online physical exercise intervention combined with online group meetings and supported by an activity watch in individuals with Type 2 diabetes. This study was designed as a one-armed feasibility study and the intervention was developed using a co-creation approach. A total of 19 individuals with Type 2 diabetes participated in eight weeks of 30 min online physical exercise intervention followed by 30 min online group meetings in smaller groups once a week. Outcomes included pre-defined research progression criteria, secondary measurements of health parameters, and participant feedback. Most research progression criteria reached a level of acceptance, with the exception of participant recruitment, burden of objectively measured physical activity, and adverse events, where changes are needed before continuing to an RCT. Combining online physical exercise with online group meetings supported by an activity watch is feasible and acceptable in individuals with Type 2 diabetes with a higher educational level compared to the general population with Type 2 diabetes.

## 1. Introduction

Engaging in physical activity behaviors is a key component in Type 2 diabetes management to improve health, quality of life, and prevent complications and premature mortality [1]. Increased physical activity is also considered a cornerstone in the treatment of hyperglycemia [1]. Hence, adults with Type 2 diabetes are recommended to engage in at least 150 min moderate- to vigorous-intensity weekly physical activity, to reduce the time spent sedentary, and break up sitting time with frequent activity breaks [1,2,3]. However, many individuals experience difficulties maintaining their motivation for physical activity [4,5]. Current physical activity interventions do not accommodate the needs of content, time, and location to preserve the individual’s daily life while increasing physical activity [6]. During recent years and throughout the COVID-19 pandemic, the use of digital information and communication technologies in healthcare has increased significantly [7]. The use of activity wearables and internet-based interventions for physical activity promotion are recommended in adults with Type 2 diabetes to adopt and maintain a physically active lifestyle [8]. Physical activity interventions delivered online (online physical exercise) could potentially meet the need of more physical activity interventions that enable the individual to attend the intervention despite a lack of resources and time, or geographical distances [6,9]. However, attending online physical exercise classes could result in a social distance causing reduced relational and mental effects and lower adherence to physical exercise sessions [9,10]. Social support is essential for individuals for managing efforts of engagement in physical activity and increase long-term adherence to physical activity interventions [6]. Combining online physical exercise with online group meetings could potentially offer health-related benefits and increase maintenance of daily engagement in physical activity through emotional and social support from individuals with similar challenges. The co-creation method involves patients in the development of health interventions, and it is an effective tool to improve adherence, satisfaction, and effectiveness [11,12]. The feasibility and effectiveness of a co-created online physical exercise intervention combined with online group meetings are yet undiscovered, although it might have a great potential to pave the way for permanent and free disease management among individuals with Type 2 diabetes. Therefore, the aim of this study was to evaluate the feasibility, fidelity, and acceptability of an 8-week high intensity online physical exercise program combined with online group meetings and supported with an activity watch in individuals with Type 2 diabetes. Outcomes included pre-defined research progression criteria, secondary subjective and objective measurements of a range of health parameters, and participant feedback.

## 2. Materials and Methods

### 2.1. Study Design

This study was designed as a one-armed feasibility study because most progression criteria were related to the received intervention. No blinding was applied in the present study. The study was carried out from 16 March to 18 May 2022 at the Centre for Physical Activity Research, Rigshospitalet, Copenhagen, Denmark. Reporting of the study followed the CONSORT extension to randomized pilot and feasibility trials [13] (Supplementary Material Table S1). This study was conducted according to the guidelines of the Declaration of Helsinki and approved by the Ethics Committee of the Capital Region of Denmark (Protocol code: H-2106295 and date of approval: 13 January 2022) and retrospectively registered in clinicaltrials.gov (NCT05668442).

### 2.2. Participants

Participants were eligible if they were above 18 years of age, diagnosed with Type 2 diabetes by a general practitioner and with access to a computer, smartphone, or tablet. The exclusion criteria were participation in another intervention study simultaneously or within the last 3 months, and if the participant’s general practitioner had advised against participation in exercise [14].

Participants were recruited from January to March 2022 from the Capital Region of Denmark and Region Zealand using several recruitment strategies such as posters on the website of the Danish Diabetes Association, via contacts from local organizations within the Danish Diabetes Association, posters in the local Diabetes Centre in Copenhagen, and lastly, participants with Type 2 diabetes from a previous trial at Centre for Physical Activity Research (CFAS) who were informed about the project through a social media forum. Potential participants underwent telephone screening with the project coordinator (MEP) to determine eligibility of participation.

### 2.3. Study Intervention

In this section, the framework of the development and design of the intervention and how it was conducted are described. First, the background for the theoretical choice of co-creation as a method with the involvement of the findings from Thorsen et al. [6], will be presented. Second, an introduction of the application of the co-creation process and a prototyping phase in practice and how it formed the actual intervention will occur.

#### 2.3.1. Development of the Intervention

The rationale of developing the intervention was inspired by the findings of Thorsen et al. [6].The study found three central themes as barriers to physical activity: (1) Physical activity conflicts with everyday life, (2) lack of physical activity opportunities and possibilities in technology, and (3) lack of community and social support. Together with the co-creation approach, these three themes created the framework for the intervention in this study. Firstly, the framework for the proposed intervention involved a physical exercise intervention that the participants could assess from their own digital device in different timeslots of a weekly day. Furthermore, the Garmin Vivofit 4 activity watch was suggested for the intervention due to its utility: small size, one year of battery life, price, and widgets. Secondly, following the online physical exercise session, it was proposed that the participants were divided into small online groups to establish relationships and social support. Lastly, the participants decided the content and agenda of the group room together. The intervention intended to initiate engagement in physical activity and support participants’ ability and motivation to increase and maintain daily physical activity by themselves.

**Figure 1:**
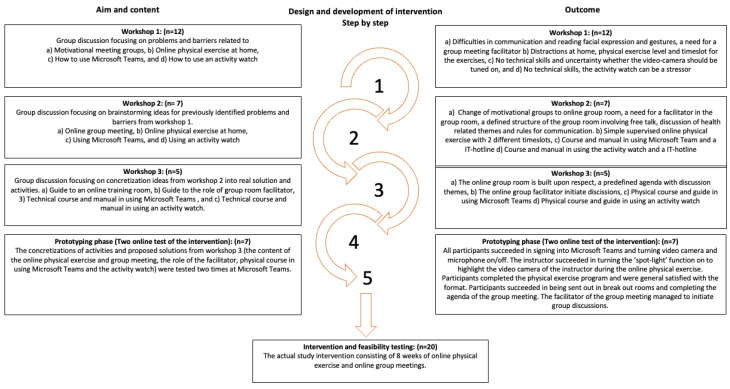
Design and development of the intervention using a co-creation approach. The development consisted of five steps: (1–3) three co-workshops, (4) a prototyping phase, and (5) the actual intervention.

Co-creation is built upon collaborations between end-users (the target population), stakeholders (people who have interest or are involved in the intervention), and academic researchers (university researchers or health-related practitioners), all working together towards a common understanding [12]. We conducted three co-workshops, a prototyping phase, and an introduction course from September 2021 to March 2022. Prior to initiating the co-creation process, stakeholders were identified via a stakeholder analysis focusing on mapping local stakeholders [15]. Stakeholders included individuals that were diagnosed with Type 2 diabetes (*n* = 12), health science researchers (*n* = 4), physiotherapists and professionals from sports science (*n* = 3), and consultants from the Danish Diabetes Association (*n* = 2). When partnership between all the stakeholders was established, information about the framework of the intervention was explained with the purpose of engaging the stakeholders in agreeing on a common goal. Afterwards, all the stakeholders were invited to a series of three face–face co-workshops. The first co-workshop focused on problem exploration, including identifying key challenges and barriers linked to the online physical exercise and community format. The following two co-workshops were inspired by “the Future Workshop” model focusing on idea generation and proposing solutions to the previously identified barriers and concretizing those ideas into real solutions and actions [16]. Afterwards, intervention content was tested in the prototyping phase with the purpose of early identification of potential issues [12]. The delivery and content of the intervention were tested using a small sample of end-users (*n* = 7) through two intervention sessions. During the test sessions, observations and feedback were obtained and refinements to the intervention were made. Following this, delivery and content were tested again. The result of the prototyping phase was the final prototype (i.e., the actual intervention) (Figure 1) [12].

#### 2.3.2. Intervention

The intervention was standardized and described in accordance with the template for intervention description and replication checklist that was developed for telehealth-interventions (TIDieR-Telehealth) [17] (Supplementary Material Table S2). As a part of the intervention, participants physically attended an introduction course before baseline testing that aimed to educate the participants in the use of Microsoft Teams, Garmin activity watches, and accelerometers.

The participants were invited to participate in 8-weeks of online physical exercise of 30 min followed by 30 min of group meeting in smaller groups once a week from March to May 2022. The participants attended the online intervention through the platform Microsoft Teams from their own devices (e.g., computer, smartphone, tablet). The intervention was scheduled on Wednesdays; one session from 10:00 to 11:00, and the other from 17:00 to 18:00. Prior to the intervention start, the participants were placed in one of the scheduled sessions as desired to accommodate other activities in their daily life.

The 30 min physical exercise program was delivered by the project coordinators (SRM (MSc Physiotherapy) and MEP (MSc Sports science and clinical biomechanics)) and consisted of a short warm-up, followed by an interval-type circuit physical exercise program consisting of bodyweight aerobic and strength exercises targeting individuals with Type 2 diabetes aimed to benefit glycemic control [8,18,19]. The interval circuit physical exercise program was intended to reach an intensity level corresponding to 16 on the Borg scale [20]. The participants were instructed in using the Borg scale, and right after the physical exercises, the participants reflected and evaluated on their reached intensity level (more detailed information about the physical exercise program is available in Supplementary Material Table S3). Following the online physical exercise, the participants were sent out in smaller predefined groups of three to five participants using the break-out room function in Microsoft Teams to conduct an online group meeting. The online group meeting served as a platform for discussion and evaluation of the online physical exercise, diabetes-related issues, and other aspects that the participants found important. Each group discussion was facilitated by a participant who had volunteered to be a facilitator prior to the intervention. The facilitators received information about how to facilitate a discussion and inspiration to the discussion topics. In addition, the facilitators were contacted by the project coordinator (MEP) by telephone after the first three online sessions to evaluate the group discussions, and afterwards the facilitators could call MEP if needed. In all other aspects, the facilitators participated in the study on equal terms in the same way as all other participants. The participants were encouraged to set personal weekly activity goals following the SMART goals structure [21] to increase self-management of habitual physical activity and evaluate their activity goals in the group discussions.

As a part of the intervention, the participants received a Garmin Vivofit 4 activity watch which they were encouraged to wear throughout all 8-weeks. Garmin activity watches were included in the study as an element to facilitate weekly activity goals and ongoing evaluation of their daily physical activity to discuss in the groups. The Garmin activity watches were chosen because they have a long battery life and a simple design to increase adherence. There were four participants that wore Garmin Forerunner 245 to compare heart rate with self-reported intensity (Borg scale) during the online physical exercise.

### 2.4. Outcomes

The outcomes included pre-defined research progression criteria, objective measurement of habitual physical activity, self-reported outcomes of a range of health parameters, and participant feedback from questionnaires. In addition, general demographic information, including age, sex, marital status, educational level, and ethnicity, and information regarding the participants’ diabetes condition, including time of diagnose, complications, medication, and HbA1c level measured by their general practitioner, were obtained from the baseline questionnaire.

#### 2.4.1. Primary outcomes

Pre-defined research progression criteria in preparation for a definitive randomized controlled trial (RCT) based on a traffic light system of green (continue without changes), amber (changes needed to improve study design and feasibility), and red (major changes are needed) [22] were the primary outcomes in this study (Table 1).

Recruitment of participants was evaluated by calculating the number of participants that were recruited within three months. Retention was evaluated by calculating the percentage of participants who provided baseline and postintervention data out of the total number of participants at baseline. To evaluate adherence to online physical exercise and group meetings, the participants received a short web-based questionnaire every week right after the online physical exercise and group meetings to respond whether they attended the sessions. Adherence was calculated by counting the number of completed online physical exercises and group meetings separately, divided by the eight planned sessions. Along with the weekly questions regarding adherence to online physical exercise and group meetings, the participants wrote down their activity goal for the forthcoming week and if they completed the activity goal from the previous week.

To evaluate improvement of habitual physical activity, all the participants were instructed to wear two Axivity AX3 (Axivity, Newcastle, UK) accelerometers for seven consecutive days before, during, and after the intervention [23]. The participants received the accelerometers by post before the measurement periods and were instructed in how to wear the accelerometers. The accelerometers were attached with a patch; one was placed on the right thigh and the other on the right side of the lower back. Accelerometer data were considered valid if the participant had minimum 22 h wear time out of 24 h that were possible. A measurement period was considered valid if the participant had at least three valid weekdays and one valid weekend day. According to the World Health Organization Guidelines on Physical Activity and Sedentary Behavior, doing some physical activity is better than none, and some physical activity will still benefit the individual’s health [2]. Therefore, any improvement in habitual physical activity (daily counts per minute) from baseline to post-intervention among participants was considered as an improvement in terms of the research progression criteria. The experienced burden of objectively measured physical activity was evaluated with a questionnaire at post-intervention regarding the participants’ satisfaction with applying and wearing the accelerometers during the intervention. The participants scored their experienced severity of adverse events in the post-intervention questionnaire following the structure of the patient-reported outcomes version of the common terminology criteria for adverse events (PRO-CTCAE^®^) [24]. In addition, the participants were told to contact the project coordinators (SRM and MEP) if they experienced any adverse events during the intervention. Minor adverse events covered dizziness, acute and prolonged musculoskeletal pain, and minor falls. Serious adverse events covered life-threatening events, disability, permanent damage, or hospitalization [25].

#### 2.4.2. Secondary Outcomes

##### Objective Outcomes

In addition to any improvement in physical activity, other aspects of habitual physical activity among the participants were explored as secondary outcomes. Activity intensity types were determined by generating ActiGraph counts using 10 s epochs from the raw acceleration data measured at the back [26]. The following activity intensity types were included: light physical activity (LPA), moderate physical activity (MPA), vigorous physical activity (VPA), moderate–vigorous physical activity (MVPA), and sedentary behavior (time spent sitting and lying) (SB). Activity intensities were additionally used to assess whether the participants adhered to the WHO recommendations of physical activity and sedentary behavior (≥150 min. MVPA or ≥75 min. VPA weekly) and the recommendations on daily physical activity according to the American Diabetes Association (ADA) and the Danish Health Authority (≥30 min. daily MVPA, which were categorized as (1) inactive day: <5 min. MVPA, (2) day with some activity: ≥5 min. and <30 min. MVPA, and (3) day with sufficient activity: ≥30 min. MVPA) [2,3]. The total daily step counts were included and determined by an algorithm by Godfrey at al. [27].

##### Self-Reported Outcomes

Baseline and post-intervention questionnaires were used to obtain secondary self-reported outcomes. The participants reported their height and weight, which was calculated into a body mass index (BMI) (kg/m^2^). Cohen’s 10-item Perceived Stress Scale (PSS) was used to assess the participants’ perceived stress, and a higher score indicated a higher perceived stress [28]. Mental well-being was assessed using the WHO5-Well-Being Index. Questions were scored from 0 (none of the time) to 5 (all of the time), and the raw scores were then multiplied by 4 to obtain a percentage score ranging from 0 to 100, where scores of ≥50 indicated a moderate to high mental well-being [29]. The Bayliss Burden of Illness Measure was used to obtain information about the number of chronic conditions and to what extent the condition interfered with daily life activities on a 5-point Likert scale of 1 (not at all) to 5 (a lot). The total scores represent the total morbidities and the total score of burden [30]. The participants’ self-perceived beliefs about their own abilities related to performing an activity were measured with the questionnaire “Self-Efficacy for Managing Chronic Disease 6-Item Scale” (SEMCD6). A higher score reflects a greater self-efficacy [31]. In addition, self-rated feelings of loneliness were assessed with the UCLA 3-Item Loneliness Scale. Each item was scored with points ranging from “hardly ever or never” (1 point) to “often” (3 points), and a higher score indicates a higher level of perceived loneliness [32].

##### Participant Feedback

The participants (both facilitators and regular participants) received a questionnaire at post-intervention about their satisfaction with the following topics: the communication between the project coordinators and participants, introduction course, online physical exercise sessions, online group meetings, setting weekly activity goals and prioritization of them, use of Microsoft Teams and Garmin watches, burden of tasks in the project, and the experience of being and having a facilitator in the online group meetings. The participants responded to what extent they agreed/disagreed with a list of statements within the abovementioned topics.

A voluntary evaluation day was held at the end of the project where the participants were encouraged to suggest potential improvements of the study design and procedures.

### 2.5. Sample Size

No sample size calculation was performed, but according to the rationale for a feasibility study, regulatory, and statistical considerations, at least 12 participants should be included to obtain a precise and representable mean and variance [33].

### 2.6. Statistical Analysis

Before the analyses were commenced, a statistical analysis plan was developed and openly available https://osf.io/3nphj/ (accessed on 14 June 2022).

Cross-tabulations were conducted to describe the participant’s characteristics. Research progression criteria were presented with descriptive statistics in accordance with the traffic light system on the per protocol population. Continuous data were presented as the mean and standard deviation or as the median and interquartile range. Categorical data were presented as the number and proportion. Changes in secondary outcomes from baseline to postintervention were reported with median and interquartile ranges (IQR) or as a number and proportions. No hypothesis-testing was carried out in this feasibility study in accordance with the CONSORT extension to randomized pilot and feasibility trials [13]. All statistical analyses were performed using Stata, Version 17, (StataCorp, College Station, TX, USA) and R statistical (R Core Team, Vienna, Austria) software version 4.2.2 (10 November 2022), RStudio (RStudio Inc., Boston, MA, USA) version 2022.07.2.

### 2.7. Deviations from the Protocol

We originally planned to collect information on adverse events at the end of every week during the intervention. However, as the participants already received several weekly questionnaires, we decided to collect detailed information about adverse events at post-intervention instead and informed participants, that they should contact the project coordinators (SRM and MEP) if they experienced any adverse events during the intervention. Furthermore, we decided not to perform a sensitivity analysis on differences in measuring daily steps between Garmin watches and accelerometers because of the small sample size.

## 3. Results

**Figure 2:**
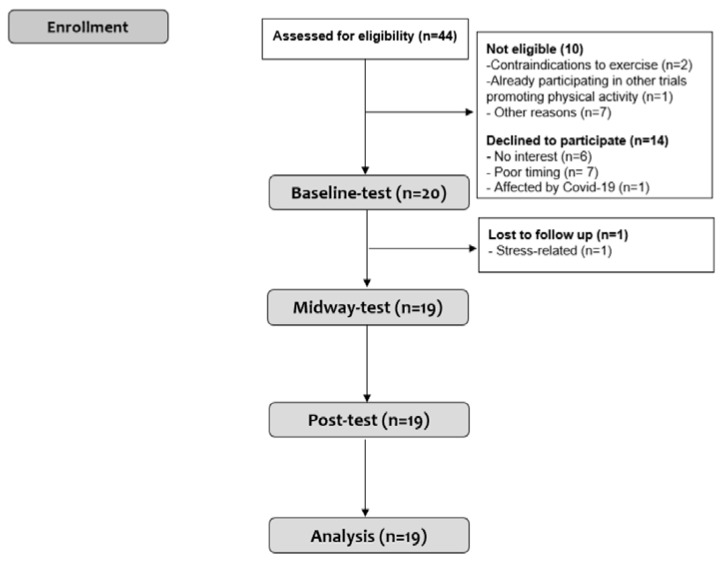
Flowchart of participant enrolment, follow-up, and analysis. Other reasons for declining to participate were stress and other mental disorders (*n* = 3), personal reasons (*n* = 1), residing abroad (*n* = 1), and loss of spouse (*n* = 1).

A total of 44 individuals were assessed for eligibility from 14 February to 10 March 2022, 20 participants were allocated to the intervention, and 19 participants were included in the analyses (Figure 2).

There were 8 females and 12 males aged 60.4 ± 8.7 and diagnosed with Type 2 diabetes that were included. Most participants had completed higher education, were overweight or obese, reported moderate perceived stress, and did not follow the WHO recommendations of physical activity (Table 2).

### 3.1. Primary Outcomes

Most research progression criteria reached a level of acceptance (i.e., green, continue to a RCT without changes), except for the criteria regarding participant recruitment, burden of objectively measured physical activity, and adverse events, which were amber (i.e., changes needed to improve study design and feasibility) (Table 3). Our target was to recruit 24 participants within three months, however, due to delayed acceptance from the Ethics Committee, we only had two months to recruit participants. Out of 19 participants, 15 (79.0%) reported that the number of days wearing the accelerometer was appropriate, which was only one percent from meeting the criterion for green. During the intervention, one participant cancelled an online physical exercise due to hospitalization with benign paroxysmal positional vertigo. The participant attended the online physical exercise the following week. Half or more of the online physical exercise sessions and group meetings were completed by 17 (89.5%) and 16 (84.2%) participants, respectively. The median [IQR] self-reported intensity (Borg scale) during the online physical exercises was 15.4 [14.4–16.8] and the median [IQR] measured heart rate with Garmin Forerunner 245 watches was 115 [111–121]. More than half of the participants with valid accelerometer data had improved their habitual physical activity from baseline to post-intervention. A total of nine participants reported minor adverse events, such as muscle pain and dizziness.

### 3.2. Secondary Outcomes

**Figure 3:**
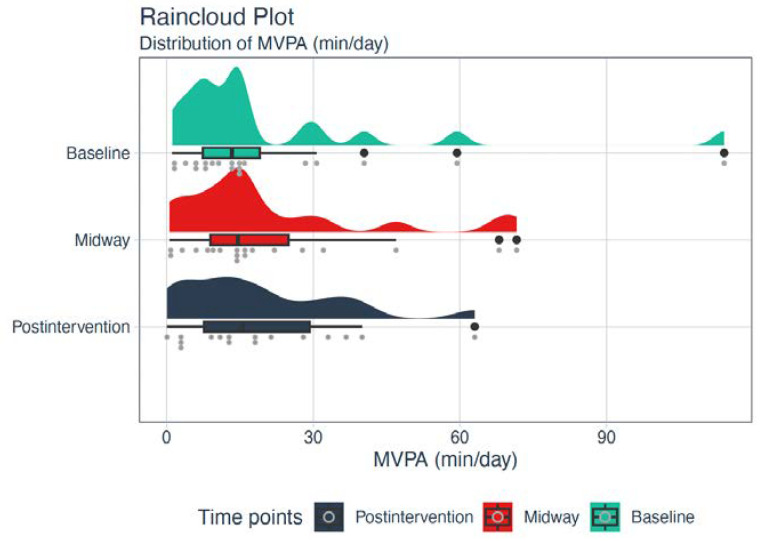
Raincloud plot representing an illustration of data in a half-density distribution (the ‘cloud’) with individual raw data (the ‘rain’) and a boxplot below the ‘cloud’ of the total daily moderate to vigorous physical activity (MVPA) in minutes at baseline, midway, and post-intervention.

**Figure 4:**
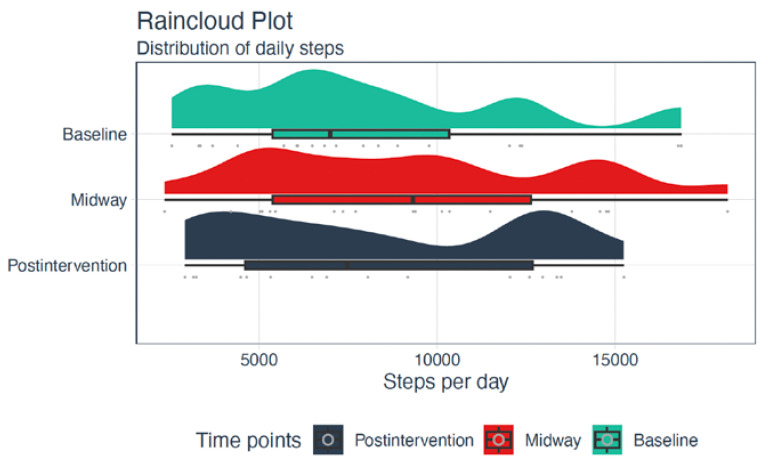
Raincloud plot representing an illustration of data in a half-density distribution (the ‘cloud’) with individual raw data (the ‘rain’) and a boxplot below the ‘cloud’ of the total daily steps at baseline, midway, and post-intervention.

The median total daily MPA, MVPA, and steps among participants increased from baseline to post-intervention (Figure 3 and Figure 4 and Table 4). Large individual differences in total daily MVPA were present among participants at baseline, which were equalized post-intervention to some degree (Figure 3). In addition, the median total daily SB decreased from baseline (10.7 h, IQR: 9.4–11.6) to post-intervention (10.3 h, IQR: 9.0–10.8). Number of days with sufficient physical activity during a week according to the recommendations from ADA and the Danish Health Authority increased from baseline (0.5 day, IQR: 0–3) to post-intervention (1.5 days, IQR: 0–3).

The median total PSS score among the participants was one point lower at post-intervention. The number of comorbidities that were reported decreased at post-intervention, however, the median disease burden that was reported increased (Table 5).

### 3.3. Participant Feedback

Most of the participants (89.5%) were satisfied with the intervention and the project met their expectations. Furthermore, most (84.2%) found it motivating to do physical exercises with others even though it was conducted online. In addition, 68.4% felt a sense of solidarity in their smaller exercise groups. Some participants (26.3%) felt that they had to prioritize participating in the study activities over other activities in their everyday life to reach their weekly activity goal (Supplementary Material Table S4).

## 4. Discussion

We found that the combination of a co-created online physical exercise intervention with group meetings supported by an activity watch in individuals with Type 2 diabetes was feasible in terms of completion of intervention, adherence to online physical exercises, group meetings, activity goals, and an improvement of physical activity. However, the data suggest that adjustments regarding participant recruitment, burden of objectively measured physical activity, and adverse events are needed before investigating effectiveness in a future RCT.

The criteria of participant recruitment did not reach an acceptable level (green) partly due to the slow processing of the project application from the Ethics Committee, which lead to a shortening of the recruitment period. Initially, the participants were recruited solely through the Danish Diabetes Association. However, due to slow rates of inclusion, participants from a previous project with a physical exercise intervention were also invited to participate [34]. Members of the Danish Diabetes Association are a selected group of Danish individuals with Type 2 diabetes, because the majority has an upper secondary or vocational education and live with their spouse [35]. The participants in this study were higher educated and had healthier lifestyle behaviors compared to the members of the Danish Diabetes Association and the general population with diabetes [35,36]. Therefore, we expect the study population to be a selected group of individuals with diabetes with higher health literacy, digital health literacy, and greater motivation for attending physical activity intervention studies [37,38]. The facilitators of the group meetings were all participants who had been engaged in either the co-creation process or the previous project with physical exercise intervention [34]. We expected the facilitators to be particularly committed and motivated to the project, which may also have influenced the other participants engagement in the project. As such, it is unclear if our intervention would also be feasible in a broader population of individuals with Type 2 diabetes with lower educational level, health literacy, and digital competences. An improved recruitment strategy is needed before proceeding to an RCT, and a new feasibility study might be required to develop a final recruitment strategy to target a wider population with Type 2 diabetes. In Denmark, general practitioners are the most frequent and first contact to the healthcare system for individuals with diabetes [39]. Recruiting participants through general practitioners could be a potential strategy to avoid a selected study population of individuals with Type 2 diabetes. Also, e-health and m-health strategies for weight loss have proven relevant and feasible in this context [40], supporting the relevance to recruit from general practitioners.

Physical exercise interventions that are delivered online are associated with more concerns about safety and adverse events due to the diminished ability for the healthcare system to take immediate action [41]. A systematic review and meta-analysis of exercise interventions delivered via videoconferencing for individuals with chronic conditions found no increased number of exercise-related adverse events and serious adverse events in the intervention groups of the included studies [41]. In our study, one serious adverse event was reported because of hospitalization with benign paroxysmal positional vertigo. However, since engagement in physical exercise is not a risk factor of developing benign paroxysmal positional vertigo [42], we do not believe that the reported serious adverse event is related to the intervention of the study. In a potential future RCT, information about adverse events should be collected weekly during the intervention period with questionnaires or text messages and via hospital records to ensure that all adverse events are identified.

The participants wore accelerometers for three periods of at least seven days before, during, and after the intervention. A total of four participants found the number of measurements days with accelerometers too burdensome. The three measurement periods were very close to each other with only few weeks in between, which could explain why some participants found the accelerometer measurements burdensome. In spite of this, the compliance was still high. A minimum of four days valid accelerometer data among adults and older adults is recommended to ensure representative data of the individual’s physical activity level [43]. A total of seven days of measurement were chosen to obtain enough valid data despite the potential occurrence of measurement errors. The study intervention was primarily focused on the individual’s own ability to increase and maintain daily physical activity, and we expected variations in physical activity from week to week. Therefore, we decided to measure physical activity three times to follow the changes before, during, and after the intervention. In a potential future RCT, the intervention period would most likely be longer than eight weeks to investigate the effectiveness of the intervention. Furthermore, the measurement during the intervention might not be needed. Hence, the measurement periods would be spread more out.

The rationale for combining online physical exercise with online group meetings and including Garmin activity watches was to accommodate the individual’s needs in terms of content, time, and location to preserve their daily life based on the findings from Thorsen et al. [6]. In addition, we intended to develop an intervention that supported the individual’s ability and motivation to increase daily physical activity by themselves. We did not expect the 30 min online physical exercise in eight weeks to increase daily physical activity significantly, but in combination with the other two components (group meetings and Garmin watches) we aimed to increase the participants’ confidence and ability to increase daily physical activity. Although limited by the small sample size and lack of control group, we found that participants increased their daily MVPA and steps from baseline to follow-up. Adherence to the online physical exercise sessions and group meetings were high among the participants, suggesting that changes in secondary outcomes were related to completion of the intervention [41]. The results of the secondary outcomes suggest that the intervention worked as intended, and participants managed to increase and maintain new physical activity behaviors in their daily life with support from their online group and activity watch. However, these findings need confirmation in an appropriately powered RCT.

This feasibility study has several limitations. Firstly, the study is limited by its design with a lack of control group and inability to ensure blinding, which precludes any firm conclusions on the effectiveness of the intervention and which components of the intervention drive our results. Secondly, the method that was used to collect information of adverse events might not have captured all adverse events during the intervention. Therefore, we cannot claim with certainty that the intervention is completely safe for individuals with Type 2 diabetes. Thirdly, the introduction course about the project was held few days before baseline measurements began, which could potentially have affected the results of baseline measurements and lead to an underestimation of the effect over time.

## 5. Conclusions

An intervention including online physical exercise, group sessions, and activity watches is feasible and acceptable for individuals with Type 2 diabetes with a higher educational level as compared to the general population with Type 2 diabetes. To claim feasibility, acceptability, and safety among the general population with Type 2 diabetes and before we can continue to a full-scale RCT, a recruitment strategy that successfully targets this population must be developed. In addition, minor changes regarding how adverse events are collected and the timing of periods with accelerometer measurements must be considered. A future RCT will demonstrate whether the intervention is also effective in increasing and maintaining physical activity in this population.

## Figures and Tables

**Table 1:** Research progression criteria for continuing to definitive RCT.

Outcome	Green	Amber	Red
Participant recruitment	24 participants recruited within 3 months	Fewer than 24 participants recruited within 3 months	Fewer than 12 participants recruited within 3 months
Completion of intervention	Minimum 75% of the participants complete postintervention	Minimum 50% of the participants complete postintervention	Fewer than 50% of the participants complete postintervention
Adherence to online physical exercise sessions ^1^	Minimum 75% of the participants complete more than half of the online physical exercise sessions	Minimum 50% of the participants complete more than half of the physical exercise sessions	Fewer than 50% of the participants complete more than half of the physical exercise sessions
Adherence to online group meetings ^2^	Minimum 75% of the participants complete more than half of the group meeting sessions	Minimum 50% of the participants complete more than half of the group meeting sessions	Fewer than 50% of the participants complete more than half of the group meeting sessions
Adherence to activity goals ^3^	Minimum 75% of the participants set goals	Minimum 50% of the participants set goals	Fewer than 50% of the participants set goals
Burden of objectively measured physical activity	Minimum 80% of the participants did NOT find the objective outcome measures of the study so difficult that they would not participate in the study again	Minimum 70% of the participants did NOT find the objective outcome measures of the study so difficult that they would not participate in the study again	Fewer than 70% of the participants did NOT find the objective outcome measures of the study so difficult that they would not participate in the study again
Improvement of physical activity ^4^	Minimum 50% of the participants have achieved improvements in physical activity at postintervention	Minimum 25% of the participants have achieved improvements in physical activity at postintervention	Fewer than 25% of the participants have achieved improvements in physical activity at postintervention
Adverse events	No or minor adverse events related to the intervention at postintervention	Fewer than five serious adverse events related to the intervention at postintervention	Five or more serious adverse events related to the intervention at postintervention

Research progression criteria based on traffic light system: Green (continue), amber (changes to protocol must be discussed before continuing), and red (do not proceed unless the issue can be solved) [22]. ^1^ At the beginning and end of the online physical exercise sessions all the participants note if they were participating. ^2^ At the beginning and end of each of the group meetings all the participants note if they were participating. ^3^ Activity goals that were assessed during the group meetings. ^4^ Any improvement in objectively measured physical activity (count per minute for the day).

**Table 2:** Baseline characteristics of participants.

Characteristics	
Age, years	60.4 ± 8.7
Women, *n* (%)	8 (40.0)
Ethnicity, *n* (%)	18 (90.0)
Living alone, *n* (%)	6 (30.0)
Educational level, *n* (%)	
Primary education	3 (15.0)
Upper secondary or vocational	7 (35.0)
Higher education	10 (50.0)
BMI, *n* (%)	
Underweight/Normal	0
Overweight	8 (40.0)
Obese Class I	8 (40.0)
Obese Class II	3 (15.0)
Obese Class III	1 (5.0)
Diet score (healthy/medium healthy/unhealthy) ^a^	7/10/3
Alcohol consumption (no alcohol/below risk group/above risk group) ^b^	7/12/1
Smoking status (smoker/ex-smoker/never smoked)	1/10/9
Adherence to WHO recommendations on weekly physical activity ^c^*, n* (%)	
Following recommendations	5 (25.0)
Not following recommendations	15 (75.0)
Adherence to recommendations on daily physical activity ^d^*, n* (%)	
Inactivity	3 (15.0)
Some physical activity	13 (65.0)
Sufficient physical activity	4 (20.0)
WHO-5-Well-Being Index total score, (0–100)	78 [72–80]
Bayliss burden of illness measure	
Median number of comorbidities reported	4 [2.5–6]
Median disease burden reported	5.5 [1.5–9]
SEMCD6, (0–10)	8 [5.7–8.7]
PSS total score, *n* (%)	
Low perceived stress	1 (5.0)
Moderate perceived stress	16 (80.0)
High perceived stress	3 (15.0)
Loneliness scale, (3–9)	3 [3–5]
Self-reported HbA1c (mmol/mol) *	47 [38–48]

*n* = 20. Data are presented as number (proportion), means (± standard deviation), or median [interquartile range]. Abbreviations: BMI: body mass index, WHO: World Health Organization, SEMCD6; Self-Efficacy for Managing Chronic Disease 6-Item Scale, PSS: Perceived Stress Scale. ^a^ Self-reported dietary habit were categorized into three items based on a diet score. ^b^ Self-reported alcohol consumption was categorized in accordance with the recommendations from the Danish Health Authority. ^c^ Adherence to recommendations on weekly physical activity according to WHO. Following recommendations of weekly physical activity: ≥150 min MVPA or ≥75 min VPA weekly or an equivalent combination. ^d^ Distribution of adherence to recommendations of daily physical activity according to ADA and the Danish Health Authority. Complete inactivity: <5 min/day of MVPA, some activity: ≥5 min/day and <30 min/day MVPA, and sufficient activity: ≥30 min/day MVPA. * *n* = 14 due to missing data.

**Table 3:** Research progression criteria results to evaluate whether to progress with a definitive RCT.

Research Progression Criteria		Evaluation
Participant recruitment, actual *n*/desired *n*	20/24	Amber
Participants who completed the intervention, *n* (%) *	19/20 (95.0)	Green
Adherence to online physical exercise sessions		
Participants who completed half of the online physical exercise sessions, *n* (%)	17/19 (89.5)	Green
Adherence to online group meetings		
Participants who completed half of the online group meetings, *n* (%)	16/19 (84.2)	Green
Adherence to goalsetting		
Participants who set activity goals, *n* (%)	19/19 (100.0)	Green
Burden of objectively measured physical activity		
Participants who did not find the attachment and shipping too time-consuming, *n* (%)	17/19 (89.5)	Green
Participants who found the numbers of days wearing the accelerometer appropriate, *n* (%)	15/19 (79.9)	Amber
Improvement of physical activity		
Participants who improved physical activity from baseline to postintervention, *n* (%) **	10/19 (62.5)	Green
Adverse events		
Participants who experienced minor adverse events, *n* (%)	9/19	Green
Participants who experienced serious adverse events, *n* (%)	1/19	Amber

*n* = 19. The research progression criteria were based on the traffic light system [22]. * 19/20 participants followed the intervention and had complete data on baseline and postintervention measurements. ** 16 participants had valid accelerometer data from baseline and postintervention.

**Table 4:** Secondary outcomes on objectively measured habitual physical activity.

	Baseline (before Week 1)	Midway (after Week 4)	Postintervention (after Week 8)
Total			
SB	10.7 [9.4–11.6]	10.2 [8.9–10.5]	10.3 [9.0–10.8]
LPA	136.8 [111.7–155.4]	133.9 [109.5–162.6]	129.2 [113.7–149.7]
MPA	9.2 [5.7–18.9]	11.7 [4.7–16.5]	12.6 [4.6–29.5]
VPA	0.3 [0.1–1.2]	0.3 [0.1–0.9]	0.3 [0.1–0.6]
MVPA	11.8 [5.8–22.2]	14.3 [7.2–19.8]	15.5 [6.2–30.5]
Daily steps	6292 [4044–9336]	8519 [5197–12068]	7479 [4569–12780]
Weekdays			
SB	11.4 [9.4–12.9]	10.6 [7.8–12.0]	10.9 [7.3–12.6]
LPA	136.8 [108.6–165.5]	131.0 [109.0–168.7]	112.3 [76.4–165.5]
MPA	7.9 [3.1–21.4]	4.8 [1.7–20.5]	7.0 [1.4–21.5]
VPA	0.2 [0–0.6]	0.2 [0–0.5]	0.2 [0–0.4]
MVPA	8.3 [3.5–23.4]	5.7 [1.8–21.7]	7.6 [1.8–22.7]
Daily steps	6620.5 [3775–9844]	7298 [3508–12273]	5910 [2709–13243]
Weekends			
SB	9.7 [6.1–11.2]	10.7 [9.3–11.6]	10.8 [9.2–11.8]
LPA	113.2 [70.7–172.3]	155.6 [118.5–195.2]	152.8 [93.7–189.3]
MPA	5.7 [0.8–11.7]	10.2 [1.8–19.8]	7.0 [3.3–33.2]
VPA	0.2 [0–0.3]	0.2 [0–0.5]	0.2 [0–0.7]
MVPA	6.0 [1.2–12.3]	11.8 [2.0–23.3]	9.3 [3.3–37.2]
Daily steps	4468 [1820–9216]	9405 [5237–14784]	9786 [4326–14252]
Adherence to WHO recommendations on weekly physical activity ^a^			
Following recommendations	4 (25.0)	4 (25.0)	6 (37.5)
Not following recommendations	12 (75.0)	12 (75.0)	10 (62.5)
Adherence to recommendations on daily physical activity during a week ^b^			
Days with inactivity	2.5 [1–6.5]	3 [1–5.5]	2.5 [1–5.5]
Days with some physical activity	5.5 [2.5–7.5]	3 [1–5]	2.5 [1–4]
Days with sufficient physical activity	0.5 [0–3]	0.5 [0–1.5]	1.5 [0–3]

*n* = 16 (participants with valid accelerometer data from baseline to postintervention). Data are presented as medians and interquartile range (IQR) (25th and 75th quartile) or *n* and proportion (%). Abbreviations: SB: sedentary behavior (hour/day), LPA: light physical activity (min/day), MPA: moderate physical activity (min/day), VPA: vigorous physical activity (min/day), MVPA: moderate to vigorous physical activity (min/day). ^a^ Adherence to recommendations on weekly physical activity according to WHO. Following recommendations of weekly physical activity: ≥150 min MVPA or ≥75 min VPA weekly or an equivalent combination. ^b^ Median [IQR] number of days during a week with inactivity (<5 min/day of MVPA), some activity (some activity: ≥5 min/day and <30 min/day MVPA), and sufficient activity (sufficient activity: ≥30 min/day MVPA) in accordance with recommendations on daily physical activity according to ADA and the Danish Health Authority.

**Table 5:** Secondary self-reported outcomes.

	Baseline (before Week 1)	Postintervention (after Week 8)
BMI	31.2 [28.7–33.7]	31.2 [28.2–32.7]
PSS total score, (0–40)	20 [18–23]	19 [17–22]
Loneliness scale, (3–9)	3 [3–5]	3 [3–5]
SEMCD6, (0–10)	8 [4.8–8.8]	8.3 [6.7–9.0]
WHO-5 Wellbeing Index total score, (0–100)	80 [72–80]	80 [72–84]
Bayliss burden of illness measure		
Median number of comorbidities	4 [3–7]	3 [2–6]
Median disease burden reported	6 [1–9]	7 [2–14]

*n* = 19 (participants with complete data on self-reported secondary outcomes from baseline to post-intervention). Data are presented as medians and quantiles (25th and 75th percentile). Abbreviations: BMI: body mass index, WHO: World Health Organization, SEMCD6: Self-Efficacy for Managing Chronic Disease 6-Item Scale, PSS: Perceived Stress Scale.

## Data Availability

The datasets that were generated and analyzed during the current study are available from the corresponding author upon reasonable request.

## Supplementary Materials

The following supporting information can be downloaded at: https://www.mdpi.com/article/10.3390/ijerph20042893/s1, Table S1: CONSORT 2010 checklist; Table S2: The TIDieR Checklist; Table S3: Description of content in the interval circuit physical exercise program; Table S4: Participant feedback.
