# Supplementary material for: Online Physical Exercise and Group Sessions to Increase and Maintain Physical Activity in Individuals with Type 2 Diabetes: A Single-Arm Feasibility Study
Source: Int J Environ Res Public Health. 2023 Feb 7;20(4):2893. doi: 10.3390/ijerph20042893 (PMC9957045; doi:10.3390/ijerph20042893)
Supplement: Supplementary file 1 [file ijerph-20-02893-s001.zip › Table S1_CONSORT checklist feasibility.pdf]

CONSORT 2010 checklist of information to include when reporting a pilot or feasibility trial*

Section/Topic	Item No	Checklist item	Reported on page No
**Title and abstract**			
	1a	Identification as a pilot or feasibility randomised trial in the title	1
	1b	Structured summary of pilot trial design, methods, results, and conclusions (for specific guidance see CONSORT abstract extension for pilot trials)	1
**Introduction**			
Background and objectives	2a	Scientific background and explanation of rationale for future definitive trial, and reasons for randomised pilot trial	1-2
	2b	Specific objectives or research questions for pilot trial	2
**Methods**			
Trial design	3a	Description of pilot trial design (such as parallel, factorial) including allocation ratio	2
	3b	Important changes to methods after pilot trial commencement (such as eligibility criteria), with reasons	2-3
Participants	4a	Eligibility criteria for participants	2
	4b	Settings and locations where the data were collected	2-3
	4c	How participants were identified and consented	2-3
Interventions	5	The interventions for each group with sufficient details to allow replication, including how and when they were actually administered	3-5
Outcomes	6a	Completely defined prespecified assessments or measurements to address each pilot trial objective specified in 2b, including how and when they were assessed	5-7
	6b	Any changes to pilot trial assessments or measurements after the pilot trial commenced, with reasons	5-7
	6c	If applicable, prespecified criteria used to judge whether, or how, to proceed with future definitive trial	5-6
Sample size	7a	Rationale for numbers in the pilot trial	7
	7b	When applicable, explanation of any interim analyses and stopping guidelines	7
Randomisation:			
Sequence generation	8a	Method used to generate the random allocation sequence	-
	8b	Type of randomisation(s); details of any restriction (such as blocking and block size)	-
Allocation concealment mechanism	9	Mechanism used to implement the random allocation sequence (such as sequentially numbered containers), describing any steps taken to conceal the sequence until interventions were assigned	-

Implementation	10	Who generated the random allocation sequence, who enrolled participants, and who assigned participants to interventions	-
Blinding	11a	If done, who was blinded after assignment to interventions (for example, participants, care providers, those assessing outcomes) and how	-
	11b	If relevant, description of the similarity of interventions	-
Statistical methods	12	Methods used to address each pilot trial objective whether qualitative or quantitative	7-8
**Results**			
Participant flow (a diagram is strongly recommended)	13a	For each group, the numbers of participants who were approached and/or assessed for eligibility, randomly assigned, received intended treatment, and were assessed for each objective	8
	13b	For each group, losses and exclusions after randomisation, together with reasons	8
Recruitment	14a	Dates defining the periods of recruitment and follow-up	4+8
	14b	Why the pilot trial ended or was stopped	-
Baseline data	15	A table showing baseline demographic and clinical characteristics for each group	9
Numbers analysed	16	For each objective, number of participants (denominator) included in each analysis. If relevant, these numbers should be by randomised group	10-13
Outcomes and estimation	17	For each objective, results including expressions of uncertainty (such as 95% confidence interval) for any estimates. If relevant, these results should be by randomised group	10-13
Ancillary analyses	18	Results of any other analyses performed that could be used to inform the future definitive trial	10-13
Harms	19	All important harms or unintended effects in each group (for specific guidance see CONSORT for harms)	10
	19a	If relevant, other important unintended consequences	-
**Discussion**			
Limitations	20	Pilot trial limitations, addressing sources of potential bias and remaining uncertainty about feasibility	14-15
Generalisability	21	Generalisability (applicability) of pilot trial methods and findings to future definitive trial and other studies	13-14
Interpretation	22	Interpretation consistent with pilot trial objectives and findings, balancing potential benefits and harms, and considering other relevant evidence	13-15
	22a	Implications for progression from pilot to future definitive trial, including any proposed amendments	13-15
**Other information**			
Registration	23	Registration number for pilot trial and name of trial registry	15
Protocol	24	Where the pilot trial protocol can be accessed, if available	15
Funding	25	Sources of funding and other support (such as supply of drugs), role of funders	15
	26	Ethical approval or approval by research review committee, confirmed with reference number	15

Citation: Eldridge SM, Chan CL, Campbell MJ, Bond CM, Hopewell S, Thabane L, et al. CONSORT 2010 statement: extension to randomised pilot and feasibility trials. BMJ. 2016;355.

*We strongly recommend reading this statement in conjunction with the CONSORT 2010, extension to randomised pilot and feasibility trials, Explanation and Elaboration for important clarifications on all the items. If relevant, we also recommend reading CONSORT extensions for cluster randomised trials, non-inferiority and equivalence trials, non-pharmacological treatments, herbal interventions, and pragmatic trials. Additional extensions are forthcoming: for those and for up to date references relevant to this checklist, see www.consort-statement.org.
