# Supplementary material for: Online Physical Exercise and Group Sessions to Increase and Maintain Physical Activity in Individuals with Type 2 Diabetes: A Single-Arm Feasibility Study
Source: Int J Environ Res Public Health. 2023 Feb 7;20(4):2893. doi: 10.3390/ijerph20042893 (PMC9957045; doi:10.3390/ijerph20042893)
Supplement: Supplementary file 1 [file ijerph-20-02893-s001.zip › Table S3_Physical exercise program.pdf]

Supplement 3. Description of content in the interval circuit physical exercise program

X_1, intended intensity (Borg scale)
X_2, number of repetitions
X_3, number of sets
X_4, number of exercises (minus warm up and cool down)
X_5, duration of each exercise (seconds)
X_6, rest in-between exercises (seconds)
X_7, rest in-between sets (minutes)
X_8, sessions per week

X_1	X_2	X_3	X_4	X_5	X_6	X_7	X_8
16	As many repetitions as possible	2	8	40	20	2	1

Exercise type	Specific exercises	Progression	Regression	Illustration
**Warm up**	Different movements and slightly increasing pace			
**Strength exercises (Set 1)**	Repeated sit-to-stand from a chair	- No support from armrests<br>- No use of chair (squats)	- Use support from armrests	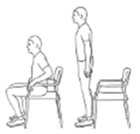
	Diagonally raises with support from a chair	- No support from chair<br>- Move opposite elbow to knee in one movement following the diagonally raises	- Raise knees supporting both hands to chair	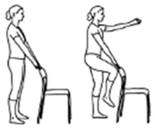
	Press ups at a wall	- Increase distance from feet to wall	- Decrease distance from feet to wall	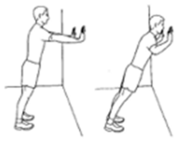
	Side flexion (sitting position)	- Raise arms<br>- Standing with wide distance between feet with knees slightly flexed – do side flexions with raised arms	- Decrease side flexion	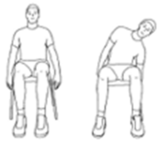
**Aerobic exercises (Set 2)**	Knee raises (standing position)	- Increase pace	- Support from a chair or wall	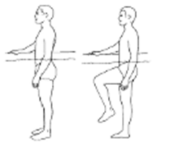

	Air-boxing	- Increase pace<br>- Do small footsteps while boxing	- Decrease pace	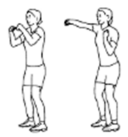
	“Twist”	- Increase pace	- Decrease pace	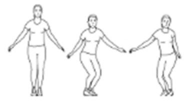
	Step sideways	- Increase pace<br>- Include arms	- Decrease pace<br>- Support from a chair or wall	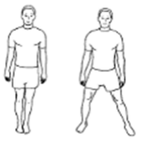
**Balance /cool down exercises**	One-leg standing	- Look in different directions<br>- Do smaller movements with arms<br>- Close eyes in small periods	- Support from a chair or wall<br>- Stand on both legs; look in different directions, do smaller movements etc.	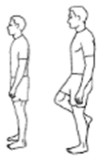
	Toe-stand	- Stand on one leg	- Support from a chair or wall	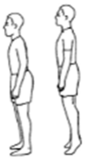
**Stretch out**	Stretch out major muscle groups			
