# Supplementary material for: Online Physical Exercise and Group Sessions to Increase and Maintain Physical Activity in Individuals with Type 2 Diabetes: A Single-Arm Feasibility Study
Source: Int J Environ Res Public Health. 2023 Feb 7;20(4):2893. doi: 10.3390/ijerph20042893 (PMC9957045; doi:10.3390/ijerph20042893)
Supplement: Supplementary file 1 [file ijerph-20-02893-s001.zip › Table S4_Participant feedback.pdf]

Supplement 4. Participant feedback

Statements	*n*, % that responded "Highly agree" or "Agree"
General satisfaction with the project	
The project met my expectations	17 (89.5)
I will recommend the project to others	19 (100.0)
I participated in the project because I wanted to	19 (100.0)
Information about the content of the project was appropriate	18 (94.7)
Participation and communication	
The communication from the project coordinator was understandable	19 (100.0)
The project coordinator was reachable during the project	18 (94.7)
The number of emails, calls and text messages was adequate	18 (94.7)
Online physical exercise	
The online physical exercise met my expectations	18 (94.7)
I felt safe turning the video camera on during the online physical exercises	18 (94.7)
The introduction to Borg-scale was appropriate	17 (89.5)
The level of the physical exercises was adjusted to my level	15 (79.0)
It was motivating to do physical exercises with others even though it was behind a screen	16 (84.2)
I will recommend the online physical exercise to others	19 (100.0)
Online group meetings	
I felt comfortable turning the video camera on during online group meetings	19 (100.0)
The group supported me to be active in every life	13 (68.4)
Being a part of a smaller online group motivated me to participate in the online physical exercises	13 (68.4)
I felt we had a sense of solidarity in the smaller online group	13 (68.4)
Activity goals	
I was able to set a realistic weekly activity goal	18 (94.7)
The smaller group supported me to reach my weekly activity goal	13 (68.4)
I had to down prioritize other things to reach my weekly activity goal	5 (26.3)
Microsoft Teams	
The introduction to Microsoft Teams was sufficient	16 (84.2)
I felt comfortable using Microsoft Teams	18 (94.7)
I experienced technical issues with Microsoft Teams	7 (36.8)
I got the necessary support and help to use Microsoft Teams	15 (79.0)
Garmin watches	
The introduction to Garmin watches was sufficient	15 (79.0)
I got the necessary support and help to use the Garmin watch	15 (79.0)
The Garmin watch's measurement of steps motivated me to be physically active	14 (73.7)
The Garmin watch was useful to set weekly activity goals	14 (73.7)
The Garmin watch was useful to reach my weekly activity goal	13 (68.4)
The role of the facilitator in the group meetings (questions only for facilitators)	
The introduction to the facilitator role was sufficient	4 (66.7)
I used the proposed agenda in the group meetings	6 (100.0)
It was time and energy consuming to be a facilitator	1 (16.7)
The role of the facilitator in the group meetings (questions only for participants)	
The facilitator had a structured agenda in the group meetings	10 (76.9)
The facilitator managed to start and direct the group discussions	10 (76.9)
The burden of tasks in the project	
The number of questionnaires was too much in the beginning of the project	2 (10.5)
The number of questionnaires was too much during the project	3 (15.8)
The number of questionnaires was too much in the end of the project	4 (21.1)
The number of smaller tasks e.g., make note of steps and intensity minutes, was appropriate	17 (89.5)
The introduction to attaching the accelerometers was sufficient	17 (89.5)
Attaching and returning the accelerometers were too time consuming	2 (10.5)
The number of days wearing the accelerometers was appropriate	13 (68.4)

n = 19

Satisfaction questionnaire from postintervention. Participants responded to what degree they agreed or not with the statements.
